# Investigation of small lung lesion detection for lung cancer screening in low dose FDG PET imaging by deep neural networks

**DOI:** 10.3389/fpubh.2022.1047714

**Published:** 2022-11-09

**Authors:** Haijun Guo, Jun Wu, Zongneng Xie, Ivan W. K. Tham, Long Zhou, Jianhua Yan

**Affiliations:** ^1^Department of Emergency Traumatic Surgery, Shanghai University of Medicine and Health Sciences Affiliated Zhoupu Hospital, Shanghai, China; ^2^Department of Nuclear Medicine, Fenyang Hospital of Shanxi Province, Fenyang Hospital Affiliated to Shanxi Medical University, Fenyang, China; ^3^Shanghai Key Laboratory of Molecular Imaging, Shanghai University of Medicine and Health Sciences, Shanghai, China; ^4^School of Medical Instrument and Food Engineering, University of Shanghai for Science and Technology, Shanghai, China; ^5^Department of Radiation Oncology, Mount Elizabeth Novena Hospital, Singapore, Singapore; ^6^Department of Radiology, Sir Run Run Shaw Hospital, Zhejiang University School of Medicine, Hangzhou, China; ^7^Department of Nuclear Medicine, Affiliated Hospital of Inner Mongolia Medical University, Key Laboratory of Molecular Imaging, Inner Mongolia Autonomous Region, China; ^8^Molecular Imaging Precision Medicine Collaborative Innovation Centre, Shanxi Medical University, Taiyuan, China

**Keywords:** lung cancer, deep learning, PET/CT, low-dose, lesion detection

## Abstract

**Purpose:**

FDG PET imaging is often recommended for the diagnosis of pulmonary nodules after indeterminate low dose CT lung cancer screening. Lowering FDG injecting is desirable for PET imaging. In this work, we aimed to investigate the performance of a deep learning framework in the automatic diagnoses of pulmonary nodules at different count levels of PET imaging.

**Materials and methods:**

Twenty patients with 18F-FDG-avid pulmonary nodules were included and divided into independent training (60%), validation (20%), and test (20%) subsets. We trained a convolutional neural network (ResNet-50) on original DICOM images and used ImageNet pre-trained weight to fine-tune the model. Simulated low-dose PET images at the 9 count levels (20 × 10^6^, 15 × 10^6^, 10 × 10^6^, 7.5 × 10^6^, 5 × 10^6^, 2 × 10^6^, 1 × 10^6^, 0.5 × 10^6^, and 0.25 × 10^6^ counts) were obtained by randomly discarding events in the PET list mode data for each subject. For the test dataset with 4 patients at the 9 count levels, 3,307 and 3,384 image patches were produced for lesion and background, respectively. The receiver-operator characteristic (ROC) curve of the proposed model under the different count levels with different lesion size groups were assessed and the areas under the ROC curve (AUC) were compared.

**Results:**

The AUC values were >0.98 for all count levels except for 0.5 and 0.25 million true counts (0.975 (CL 95%, 0.953–0.992) and 0.963 (CL 95%, 0.941–0.982), respectively). The AUC values were 0.941(CL 95%, 0.923–0.956), 0.993(CL 95%, 0.990–0.996) and 0.998(CL 95%, 0.996-0.999) for different groups of lesion size with effective diameter (R) <10 mm, 10–20 mm, and >20 mm, respectively. The count limit for achieving high AUC (≥0.96) for lesions with size *R* < 10 mm and *R* > 10 mm were 2 million (equivalent to an effective dose of 0.08 mSv) and 0.25 million true counts (equivalent to an effective dose of 0.01 mSv), respectively.

**Conclusion:**

All of the above results suggest that the proposed deep learning based method may detect small lesions <10 mm at an effective radiation dose <0.1 mSv.

**Advances in knowledge:**

We investigated the advantages and limitations of a fully automated lung cancer detection method based on deep learning models for data with different lesion sizes and different count levels, and gave guidance for clinical application.

## Introduction

Lung cancer is still the leading malignant disease for women and men (1). Low dose computed tomography (CT) was recommended for lung cancer screening for high-risk populations. Positron Emission Tomography (PET) with 18-fluorodeoxyglucose (FDG) has been widely used in the management of lung cancer (2). In addition, FDG PET/CT imaging is often recommended for the diagnosis of pulmonary nodules after indeterminate low dose CT lung cancer screening (3), especially for small nodules <10 mm. However, since *γ*-ray radiation associated with PET imaging may carry health risk for patients, lowering PET tracer injection is highly desirable. However, reducing tracer dose within the current imaging protocols and reconstruction settings will inevitably lead to poor image quality such as low signal-to-noise ratio (SNR) or low contrast-to-background ratio, which may cause unreliable diagnosis (4). Besides, the interpretation of FDG PET/CT images is more challenging for low SNR images, especially for less-experienced physicians. Given wide variations of interpretations of images leading to different diagnoses and being a laborious task which may be translated into high costs and human errors, health professionals can benefit from computer-assisted interventions. Computer assisted diagnosis based on machine learning could help minimize the errors or improve the clinical management.

Recently, deep learning (DL) has established noteworthy progress in computer vision (5) and now attracted increasing attention in medical imaging (6), including image classification, object detection, registration, image denoising, segmentation and image modality transaction (7, 8). All these technical development could be translated into clinical benefit including improve diagnosis, increasing working efficiency and standardizing the protocol. The power of DL has been demonstrated in the lung node or lung cancer detection based on low dose CT images (9). There are less similar works for FDG PET imaging. Poor quality of low-dose PET images due to reduced injection dose limits the use of deep learning models in low-dose PET images. Schwyzer et al. (10) evaluated deep learning for the detection of lung cancer in simulated low-dose FDG-PET imaging. Sibille et al. (11) trained a convolutional neural network (CNN) on PET/CT data to classify the malignancy of lymphoma and lung cancer. Teramoto et al. (12) proposed to use a CNN to improve false positive reduction for the detection of pulmonary nodules in PET/CT images. Abnormality detection is another effective way, which only requires data with labeled by radiologists as either normal or abnormal.

In the similar work (10), Schwyzer et al. firstly investigated the utility of deep learning method in abnormality nodule detection with FDG PET imaging and tested the performance with lowering dose. Fully automated lung cancer detection can be realized at a very low effective radiation dose of 0.11 mSv. However, in that work, PET images were converted to PNG format before training the deep learning model, which may lose much information. In addition, the dose limit for the detection of different size lesion was not explored.

Inspired by the previous work (10), we fine-tuned the commonly used ResNet50 on the original DICOM data to detect lung nodule and investigated the benefits and limitations of this approach on different count level data for different lesion size.

The rest of paper is organized as follows. Section 2 introduces the methodology and experimental setup followed by the results of Section 3. Finally, we discuss and presents a concluding summary of this work in Section 4.

## Methods

### Network

We used a 50-layer convolution neural network to for the nodule diagnosis. The network used a ResNet50 architecture (13), which won first place on the 2015 IMAGENET Large Scale Visual Recognition Challenge (ILSVRC) classification task (14). The main reason for choosing the residual network was to build a deeper neural network without reducing accuracy. In order to adapt for our purpose, we replaced the final fully connected layer with one that has a single output, followed by a sigmoid activation function.

For each image *X* of study type *T* in the training set, we optimized the binary cross entropy loss


(1) L(X,y)=−y log(p(Y=1|X))−(1−y) log(p(Y=0|X))

Where *y* is the label of the study, *p*(*Y* = *i|X*) is the probability that the network assigns to the label *i*.

Before feeding images into the network, we scaled the variable-sized images to 64 × 64 and augmented the data during training by applying random lateral inversions and rotations of up to 90 degrees. The weights of the network were initialized with weights from the ResNet50 model pre-trained on ImageNet dataset. The network was trained end-to-end using Adam (15) with default parameters *β*_1_ = 0.9 and *β*_2_ = 0.999. A mini-batches of size 16 was used. The initial learning rate was set to 0.0001, after *n* epochs, a warm restart was performed where the learning rate was reset to its initial value and the optimizer's momentum buffers were cleared creating a temporary period of instability allowing the optimizer to escape sub-optimal local minimums (16). All experiments were conducted using the Keras (17) with Tensorflow backend on a NVIDA TITAN GTX GPU.

### Data pre-processing and experiment settings

Twenty patients (weight 37.2–91 kg) with biopsy-proven primary lung cancer were used for this study. All scans were performed on a Biograph mCT (Siemens Healthcare Molecular Imaging) after an uptake period of 60 min with injection of 218.3 ± 5.18MBq FDG. The PET images were acquired in list-mode format, and the true scan counts were obtained by subtracting the smoothed delayed counts from the total prompts (the true and scattered events). All patients were scanned with 2 bed positions covering the lung for 10 min, resulting in 120 ± 25 million mean true coincident counts per bed position (18). Low dose PET images were simulated by randomly discarding events in the PET list-mode data to obtain 9 different predefined true count levels with 20 × 10^6^, 15 × 10^6^, 10 × 10^6^, 7.5 × 10^6^, 5 × 10^6^, 2 × 10^6^, 1 × 10^6^, 0.5 × 10^6^ and 0.25 × 10^6^ counts. The reconstruction was Ordinary Poisson Subset Expectation Maximization (OP-OSEM), using Time of Flight (TOF) and Point Spread Function (PSF), with 2 iterations, 21 subsets and 3 mm Gaussian filtering. The size of each reconstructed 3D PET data is 400 × 400 × 171 (2.04 × 2.04 × 2.03 mm).

**Figure 1:**
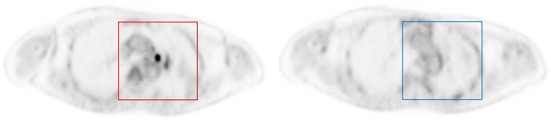
Example of image patches with lesion **(left)** and background **(right)**. Each patch was annotated to either 0 if no nodule was present (blue square), or 1 if an FDG-avid nodule was present (red square).

The data were divided into training (12 patients), validation (4 patients) and testing (4 patients) subsets. The validation was used to guide the algorithm development and to select the algorithm hyper-parameters, while the test set was exclusively used to assess the performance. The lesion and background images patch of PET images were chose by an experienced physician and served as ground truth for the ResNet50. A total of 1,145 PET image patches (64 × 64) were obtained. In order to study the performance of deep learning model at different count levels, a total of 10,307 PET image patches from 9 different count levels at the same position as annotated were obtained. At the same time, a total of 11,307 background patches in all 9 count levels were randomly cropped in normal organ tissue with fixed patch size as same as lesion patches. We produced SUV (Standardized uptake value) image *via* Eq. 2, rather than converting to PNG image. Each patch was associated with either a label 1 (pulmonary nodule is present on the patch) or a label 0 (background), as mentioned above and illustrated in Figure 1.


(2) $$\begin{array}{l} \text{SUV}=\;\frac{{\text{C}}_{\text{img}}\left(\text{t}\right)}{\text{ID}/\text{BW}} \end{array}$$

Where *C*_*img*_(MBq/mL) is radioactivity concentration, *ID*(MBq)is the injected dose at *t* = 0, and *BW*(kg) is the body weight.

### Statistics analysis

For the calculation of the ROC curves and AUC value, we used the scikit-learn package (19) and Bootstrap method to estimate confidence level.

## Results

**Figure 2:**
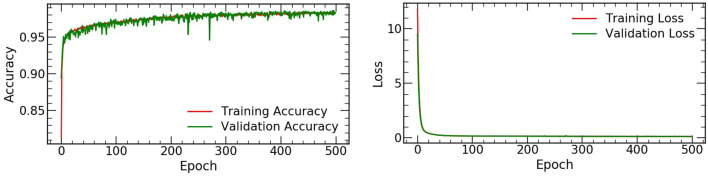
The accuracy and loss values on the training and validation datasets.

The accuracy and loss values on the training and validation datasets were shown in Figure 2, and the performance of the automated detection lung cancer was evaluated by using AUC. A total of 3,312 and 3,384 patches for lesion and background were chosen from 4 test patients at the all count levels, respectively. For each lesion patch, we generated ROI in the full-dose images by selecting all pixels within the annotated location with values equal to or greater than threshold of 40% of the maximum SUV value. The size of lesion was estimated by the effective diameter (defined by ourself) by converting the lesion area to diameter of a circle, and the number of different lesion sizes were summarized in Table 1.

**Table 1:** The total number of image patches with different lesion sizes for the test (summation of all 9 count levels).

R [mm]	[0, 10]	[10,20]	[>20]
Number of patches	549	1,143	1,620

### Count level study

**Figure 3:**
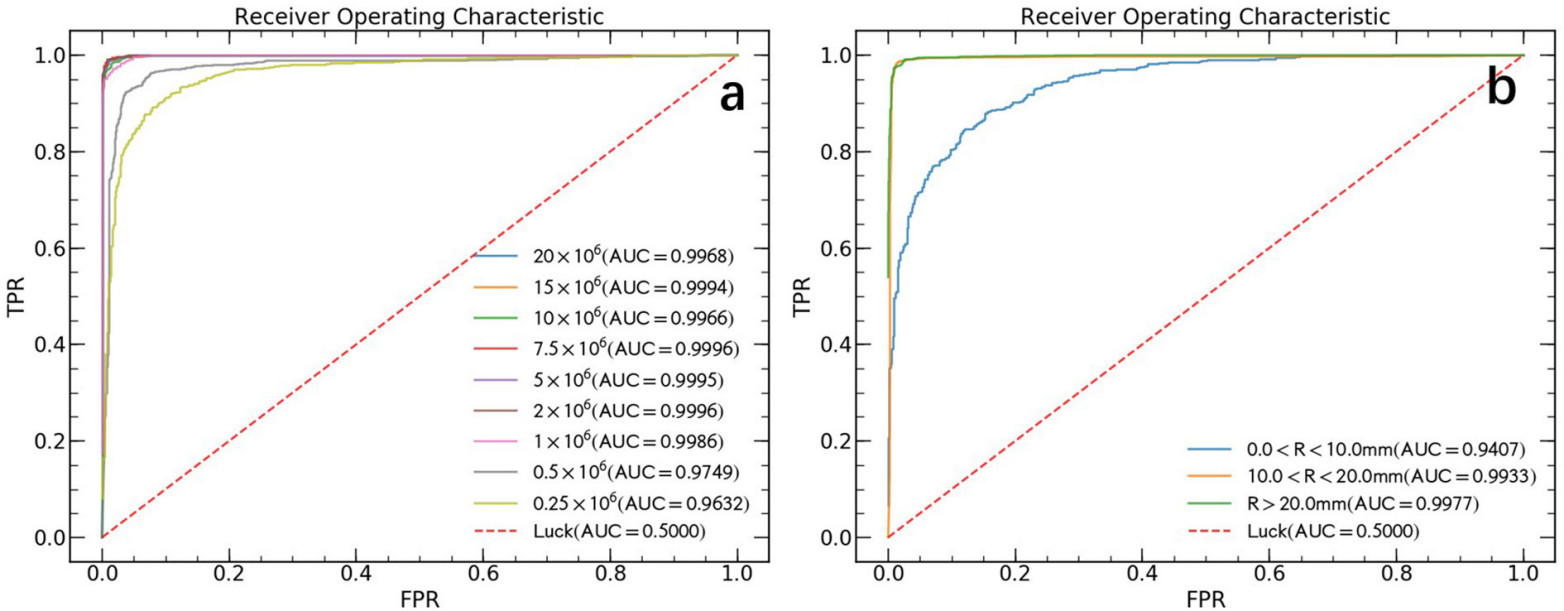
ROC curves in the test data set. **(a)** The ROC curves of different count levels. FPR and TPR refer to false positive rate and true positive rate, respectively. **(b)** The ROC curves for lesions of different sizes without distinguish between count levels. Where *R* represents the effective diameter of lesion and calculate by converting the lesion area to diameter of the circle.

The ROC curves comparison between all 9 different count levels of PET images are illustrated in Figure 3a. In general, the AUC values decrease as true count decreases and rapidly reaches saturation after a count level >1 × 10^6^. Derived from the AUC, the sensitivity and specificity for automated lung cancer detection were 96.7 and 98.5% for true count 1 × 10^6^, 92.9 and 94.9% for true count 0.5 × 10^6^ and 83.3 and 99.2% for 0.25 × 10^6^, respectively.

### Lesion size study

False negative findings are mainly represented by small lesions (<10 mm) al- though 18F-FDG PET sensitivity may be reduced in specific tumor types showing variable FDG uptake (2). Previous paper (10) also pointed out, their high false negative rate (FNR) can be explained by lower tumor activity and small lesion size (< 10 mm). In this study, we made an analysis of our model performance on different lesion sizes data and the results are shown in Figure 3b. We found that the AUC value increased with increasing lesion size and rapidly reached saturation. The final obtained AUC values are 0.941 (CL 95%, 0.923–0.956), 0.993 (CL 95%, 0.990–0.996) and 0.998 (CL 95%, 0.996–0.999) for lesion size with effective diameter (R) < 10 mm, 10–20 mm, and > 20 mm, respectively.

### Detectability

In order to systematically study model performance and to survey detectability of lung cancer lesions, we analyzed the dependence of model performance with AUC values on all 9 count levels and three different lesion sizes data. The analysis results are shown in Table 2. The AUC value increased rapidly and reached saturation with the increase of lesion size and true count. For lesions with size larger than 10 mm, the model can achieve a AUC ≥ 96% for all count levels, even at the count level of 0.25 × 10^6^ counts. At least 1 × 10^6^ counts were required to achieve the AUC larger than 0.95 for the lesion with <10 mm.

**Table 2:** The AUC value for lesions with different lesion size and true count level.

R [mm]\Count [1*e*^6^]	0.25	0.50	1.00	2.00	5.00	7.50	10.00	15.00	20.00
10									
	0.503	0.798	0.951	0.983	0.979	0.996	0.987	0.983	0.986
10,20	0.985	0.964	0.999	0.999	1.000	0.999	0.996	0.999	0.996
>20	0.960	0.995	1.000	1.000	1.000	1.000	1.000	1.000	1.000

The bold values represent AUC < 0.96 and maximum improvement, respectively.

### Ablation study

We performed an ablation study to understand the effectiveness of our designs using SUV scaling. All the experiments were conducted with the same dataset so that both the quantitative and qualitative performances can be evaluated. Table 3 shows the experimental results, where the performances of deep learning model with and without SUV scaling are compared. The test results are presented by relative AUC improvement (*AUC*_*imp*_), where the baseline is the model trained without SUV scaling data, and we observed 4.7% improvement for small size lesion (*R* < 10 mm).

**Table 3:** The AUC improvement via SUV scaling.

R [mm]	[0, 10]	[10,20]	[>20]
*AUC_*imp*_*	4.7%	0.4%	0.2%

The bold values represent AUC < 0.96 and maximum improvement, respectively.

### Model interpretation

We visualized the parts of the PET images which contribute most to the model's prediction of lung cancer by using class activation mappings [CAMs (20)]. We input a PET image *X* into the fully trained network to obtain the feature maps output by the final convolutional layer. To compute the CAM *M* (*X*), we took a weighted average of the feature maps using the weights of the final fully connected layer. Denote the *k*th feature map output by the network on image *X* by *f*_*k*_(*X*) and the *k*th fully connected weight by *w*_*k*_. Formally,


(3) $$\begin{array}{l} M\left(x\right)=\;\underset{k}{\sum }w_{k}f_{k}\left(x\right) \end{array}$$

**Figure 4:**
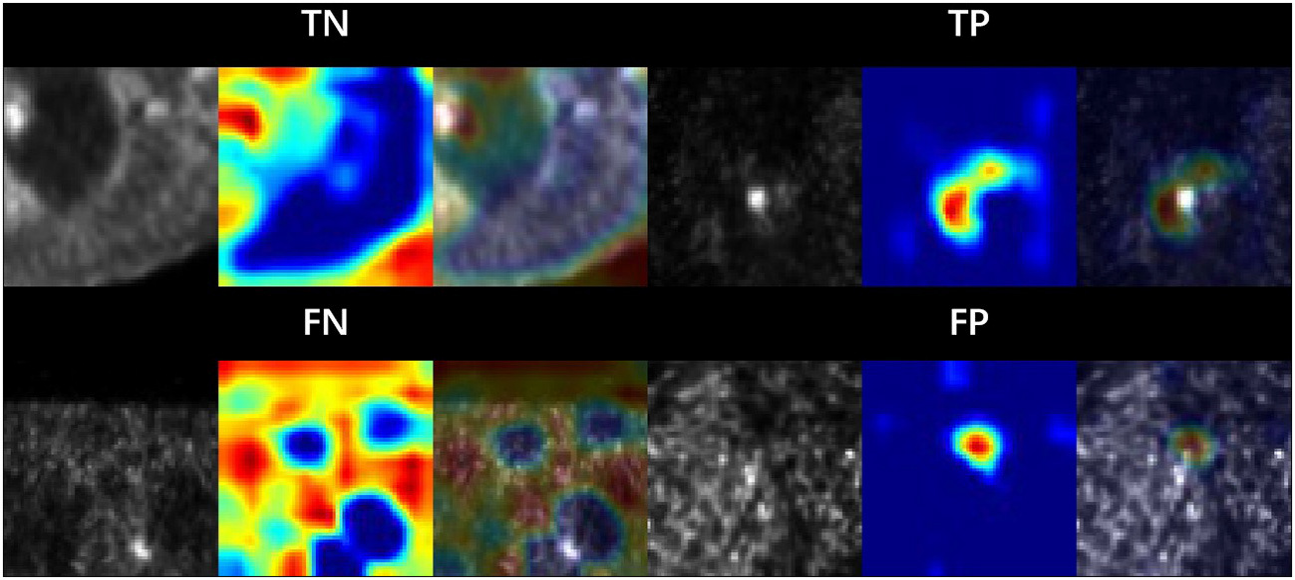
Sample patches with different categories. The area is highlighted by the model CAM. Where TP, TN, FP and FN are corresponds to true positive, true negative, false positive and false negative, respectively.

To highlight the salient features in the original PET image, which contributed the most to the network predictions, we upscaled the CAM *M* (*X*) to the dimensions of the image and overlay the image. Figure 4 shows the example PET images and the corresponding CAMs output. The activation maps for false negative (FN) and false positive (FP) cases revealed that noise, low tumor activity and small lesion size are the main causes.

## Discussion and conclusion

We showed that a larger lesion size and smaller noise level (larger true counts) for PET images performs better than a smaller lesion size and larger noise level (smaller true counts) for most of the lung cancer detection, which can be explained by more discriminative features that are crucial for detection and classification. In order to detect small size lung cancer, we made several efforts, including new pre-process, e.g. converting radioactivity value to SUV scale, and using stochastic gradient descent with warm restarts (SGDR) (16) strategy to improve model optimization during training. Besides, we made a systemic study of model performance with a series of different lesion size data and different true counts data in PET images. We observed the AUC value increased rapidly and reached saturation with the increase of lesion size and true count.

Although the results are promising, there still exist some limitations. Firstly, the low-dose PET images are produced base on a simulation, even though recent work (21) confirmed that the emulated low-count scans are comparable to low-dose scan. It is understood that real low-dose images might be somewhat different from simulated low-dose images. This is due to potentially different biodistribution and different count statistics. Secondly, due to the limited collected PET/CT images, we only used 2D CNN network to detect lung cancer patch-by-patch. When we apply it into clinic, we need to perform sliding window detection, which may affect the detection efficiency. Finally, since lung cancer have a 3D structure, a 3D convolution would be the more reasonable option. We also note that deep learning has a wide range of applications in low-dose PET/CT image noise reduction (22–24). If we combine the deep noise reduction model with the lesion detection model, it will help to improve the detection performance of the model at low doses. In addition, combining PET and CT images would further improve the model performance and reduce the false-positive rate (12). In the future, we will collect enough annotated data and consider using 3D CNN network to detect lung cancer as well as combining noise reduction in low-dose PET/CT images to improve detection performance and robustness.

The potential of lung cancer detection based on deep learning in low-dose PET imaging has not been widely explored. The recent published paper (10) on lung cancer detection shows good performance, but they still didn't investigate the robustness to count level and lesion size. In addition, they directly convert the PET images to PNG images during image preprocessing by scaling the maximum value in PET image to 255. However, the maximum value in the PET images are not stable and are largely affected by noise, especially at low-dose PET images. Such a conversion would lead to a significant difference in pixel values of similar lesion in PNG images. As shown in Eq. 2, it is defined as the ratio of activity per unit volume of a region of interest (ROI) to the activity per unit whole body volume and is considered to be a semi-quantitative parameter. Abnormal SUV values are an important feature for lung cancer, and this conversion leads to loss of SUV information. In addition, our ablation study also confirmed that the SUV scaling aids in the detection of small lesions.

Lung cancer detection at low-dose PET/CT image has important clinical applications: reading workflow could potentially be simplified by preprocessing PET/CT to more efficiently derive clinically relevant parameters such as automatic parametric lesion description. Besides, automated lung cancer detection could help combat radiologist fatigue. Physician fatigue is a common problem that affects all healthcare professionals, radiologists are particularly susceptible (25). Thus, a model which can perform automatic lung cancer detection and localization could highlight the portion of the image that is recognized as abnormal by the model, drawing the attention of the clinician (26). In the future, we will test the model with prospective study and introduce this approach into the current clinical workflow.

In conclusion, we proposed a deep learning method that could automatically diagnose pulmonary nodule and explored its performance with different lesion size at the different count level.

## Data availability statement

The raw data supporting the conclusions of this article will be made available by the authors, without undue reservation.

## Ethics statement

The studies involving human participants were reviewed and approved by Domain Specific Review Board of National Healthcare Group, Singapore (Ref: 2014/00459). The patients/participants provided their written informed consent to participate in this study. Written informed consent was obtained from the individual(s) for the publication of any potentially identifiable images or data included in this article.

## Author contributions

HG, JW, ZX, and LZ: conceived and designed the study, acquired data, and conducted statistical analyses and interpretation. HG, JW, and LZ: drafted the manuscript and provided statistical support. HG, JW, IT, and JY: provided professional support, and made several critical revisions to the manuscript. All authors read and approved the final manuscript.

## Funding

This study has received funding by the National University Cancer Institute, Singapore Centre Grant Seed Funding Program, the National Natural Science Foundation of China 81671775.

## Conflict of interest

The authors declare that the research was conducted in the absence of any commercial or financial relationships that could be construed as a potential conflict of interest.

## Publisher's note

All claims expressed in this article are solely those of the authors and do not necessarily represent those of their affiliated organizations, or those of the publisher, the editors and the reviewers. Any product that may be evaluated in this article, or claim that may be made by its manufacturer, is not guaranteed or endorsed by the publisher.
